# Quantifying shrub encroachment through soil seed bank analysis in the Ethiopian highlands

**DOI:** 10.1371/journal.pone.0288804

**Published:** 2023-08-21

**Authors:** Shambel Alemu Chengere, Cara Steger, Kflay Gebrehiwot, Sisay Wube, Bikila Warkineh Dullo, Sileshi Nemomissa

**Affiliations:** 1 Department of Plant Biology and Biodiversity Management, College of Natural and Computational Sciences, Addis Ababa University, Addis Ababa, Ethiopia; 2 Department of Natural Resources and Environment, Cornell University, Ithaca, NY, United States of America; 3 Department of Biology, Samara University, Semera, Ethiopia; 4 Applied Behavioural Ecology and Ecosystem Research Unit, School of Ecological and Human Sustainability, University of South Africa, Florida, South Africa; 5 Forest and Rangeland, Ethiopian Institute of Biodiversity, Addis Ababa, Ethiopia; Universidade Federal de Minas Gerais, BRAZIL

## Abstract

This study aimed to understand the impact of shrub encroachment on native species in the Guassa Community Conservation Area in Ethiopia. We assessed the soil seed bank composition and density across different elevations and aspects, and management systems within the area. The vegetation was stratified and eight blocks were selected across a range of elevation (<3350 m and >3350 m) and aspect (northeast, northwest, southeast, southwest). Within each block we established twenty 5m x 5m plots for a total of 160. We then collected soil samples from five subplots (1 m x 1 m) at three depths (0–3 cm, 3–6 cm and 6–9 cm) for a total of 480 samples, which were established in pots in greenhouse. We calculated species abundance by totaling the number of seedlings that emerged from each sample. To determine the variability in the abundance of *Festuca macrophylla* and *Helichrysum splendidum* in the soil seed bank along altitudinal gradient, we used two-way ANOVA using SAS statistical software version 9.0.1. Shannon diversity index was used to determine species diversity in the soil seedbank. After counting all the seeds, we identified 74 plant species represented in the soil seedbank which belong to 55 genera and 23 families. Eleven species are endemic to Ethiopia. At the lower elevation range, the effects of aspect (P <0.0088) and soil depth (P <0.005) are not significant to determine the abundance of seeds of *H*. *splendidum* and *F*. *macrophylla*. But when the factors are segregated, both aspect and soil depth play a significant role (p<0.0001) regarding the abundance of the seeds of the competing species at lower elevation. At higher elevation, only the effect of soil depth is significant (P<0.0001) for determining the abundance of *H*. *splendidum*. Soil depth and aspect have no significant effects on soil seed bank abundance at this elevation.

## 1. Introduction

Ethiopia is a country located in the Horn of Africa, characterized by a wide variety of landscapes such as rugged mountains, river valleys, flat-topped plateau, deep gorges, and rolling plains. The country is also known for its vast wealth of natural resources and biological diversity, which spreads across higher altitude areas, from mountain peaks to one of the lowest and hottest places on earth. The country is also the second most populous in Africa experiencing a major land cover change [1, 2]. The land cover changes in the country are mainly taking place from lands with natural vegetation to agricultural lands and residential area. Several studies [1, 3–7] show that Ethiopia’s ecosystems including forest, woodlands, grasslands and shrub lands are rapidly declining while farmland and bare land are expanding over time. The leading factor for these is population growth which resulted in competition for natural resources, pervasive poverty, food insecurity, high unemployment rate, drought and other socio-political factors [4, 6, 8]. These has led to rapid expansion of agricultural land and expanding bare land at the expense of the loss of native vegetation. Comparing land cover in the high and low lands of Ethiopia, the changes in ecosystem are more severe in the highlands of Ethiopia [9]. This is because of high population pressure, shrub expansion and long-term cultivation on the highland areas [1].

From the above factors, shrub expansion into grasslands is reported worldwide [10, 11], affecting both low and highland grasslands [12, 13]. Especially shrub expansions in the highlands are being reported by different scholars leading to ecosystem service changes. This vegetation change has been attributed to climate change, atmospheric CO_2_ concentrations, nitrogen deposition, and changes in local management practices such as agricultural expansion, cutting, grazing pressure or increased fire frequency [10, 14, 15]. The transition from grassland to an ecosystem dominated by woody species could lead to dramatic changes in community structure and function, such as reduced species richness and diversity [16] and risk increased fire [17].

The shift from a grass-dominated to a shrub-dominated ecosystem is likely to have a significant impact both on plant productivity and location due changes in the distribution of net primary productivity (NPPs) between aboveground and belowground components [18]. Consequently, grasses have a high distribution of NPPs below ground, while shrubs may release more NPPs above ground [19]. The increase in aboveground NPP due to shrub encroachment is due to the potential of shrubs to support a much larger leaf area than grasses under similar climatic and resource constraints [20].

Shrub encroachment is common in most arid and semi-arid biomes of the world. For instance, the transformation of large areas of former black grama pastures (*Bouteloua* spp.) in dense thickets of bush lands [21], of the United State was caused by the dramatic increase in density of mesquite (*Prosopis glandulosa*) and creosote bush (*Larrea triangotensi*). Increased abundance of shrubs has been a major problem for African pastures used for livestock [22]. In South Africa, for example, 13 million hectares are under bush encroachment and it is estimated that, together with the loss of savannah systems, more than two billion people worldwide will be affected [23]. In East Africa, a 10% increase in bush area reduces grazing by 7%, and grazing is completely eliminated at 90% bush cover [24]. In Ethiopia, the Borana rangelands, the encroachment of shrubs and unpalatable forbs threatened about 83% of the rangelands [25]. Furthermore, in the Nechisar National Park shrub encroachment deteriorated herbaceous species cover-abundance [26].

Guassa Community Conservation Area, one of the Afroalpine grassland, is characterized by rich biodiversity and ecosystem services in Ethiopian highlands. It is a home to a large amount of fauna and flora [27]. The local community living around guassa area traditionally managed and uses the resource for various purposes. For example, the area provides services such as livestock grazing, as a firewood, and cutting guassa grass (*Festuca macrophylla*) which uses for various purposes such as thatching, making household equipment, baskets, painting brushes, mattresses and shepherds’ raincoats and farm implements like ropes and whips. At present, nine farmers’ associations collectively manage an area recognized by the Amhara National Regional State as the Guassa Community Conservation Area (GCCA). This area plays crucial role for the livelihood and coping strategies of the community living around the area, especially during periods of drought, by providing food for animals. On the other hand, the local community uses different shrubs mainly *Helichrysum splendidum* as wood for cooking and for keeping their households warm during cold time [28].

However, this Afroalpine region which has global and local importance is under increasing threat from high rate of expansion of *H*. *splendidum* shrub and its encroachment over other habitats like *Festuca* grassland. *H*. *splendidum* is a fast-growing shrub; it can grow to 1.5 m x 1 m within 2 years, forming a dense grey mound. This easy to grow shrub requires very little maintenance provided that it is given a large enough area to spread. On the study area, according to the local community and other stakeholders (like FZS, local government bodies), the spread of this species is fast and it threatens the local biodiversity and their livelihood. The high rate of expansion of *H*. *splendidum* (due to climate change, temporary drought, and human disturbance while harvesting guassa grass) [29] and its encroachment over other habitats like *Festuca* grassland was also pointed out [30]

The assessment of soil seedbank is very crucial for understanding the competitive ability of species, conservation planning and management of species composition and structure. Studies shows that soil seedbank provide a viable option for restoration through the establishment of native vegetation [31]. In Ethiopia, particularly in our study area, there are only scant information concerning soil seedbank [32, 33], especially for the two dominant plant species (*Helichrysum splendidum* and *Festuca macrophylla*) hereafter called the target species. This study is designed based on our observation of the current management system of the Guassa Community Conservation Area, which seems to favor the encroachment of *Helichrysum splendidum* as compared to *Festuca macrophylla*. In addition, our previous research has shown that [34–36], the local community perceives the encroachment of *Helichrysum splendidum* as sustainability threat. These two species are by far the most dominant species in the area. However, there is a general perception that the grass (*Festuca macrophylla*) is losing competition to the shrub (*Helichrysum splendidum*). The current management of system of the area involves harvesting *Festuca macrophylla* every two-three-year cycle before the grass set seeds because the vigor of the grass decreases upon flowering [30]. Therefore, based on this observation and our previous finding, we set to find out if there is a significant difference in terms of soil seedbank density between these two species. However, the studies of soil seedbank in a conservation area where the diversity and composition of species are high unavoidably involve other species in addition to the target species. Therefore, we addressed the following questions with an objective to assess whether the management system being implemented in the conservation area is favoring one target species over the other. a) What is the status of soil seedbank of GCCA in terms of species diversity, composition and seedbank density? b) Does the soil seedbank density of *Helichrysum splendidum* and *Festuca macrophylla* species vary along aspect and altitudinal gradients? c) Does the management systems gave *Helichrysum splendidum* a competitive ability to encroach into *Festuca macrophylla* stands?

## 2. Materials and methods

### 2.1. Study area

**Fig 1:**
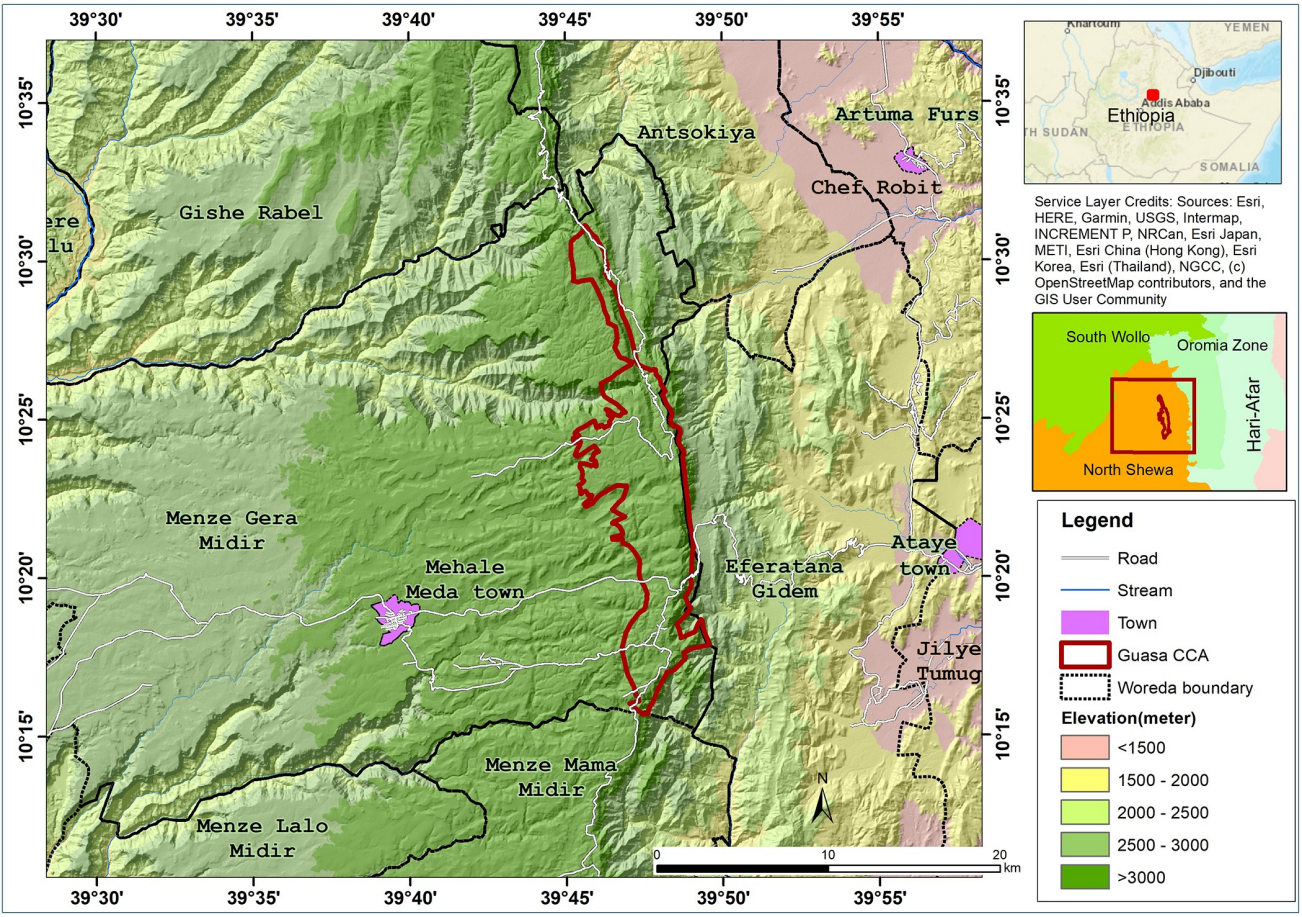
Map of Ethiopia showing the Guassa Community Conservation Area (GCCA).

Ethiopia is one of the 25 biodiversity-rich countries in the world, where two of the 36 biodiversity hotspots in the world [37, 38], namely the East Afromontane and the Horn of Africa [39], trespass through its mountain and lowland areas. Ethiopia consists of three major high plateau regions divided by the Great Rift Valley and by the Abay (Blue Nile) Gorge. These three highland blocks are known as the north-western highlands, the central highlands and south-eastern highlands. The north-western highlands are the largest highland massif. The GCCA is located in the central highlands at 10°15’–10° 27’ N and 39° 45’–39° 49’ E and is situated in an area locally known as Menz Gera Midir district [40]. It is found in the Amhara Regional State of North Shewa, 295 km north-east from Addis Ababa, capital city of Ethiopia (Fig 1). The guassa range comprises 111 km^2^ and lies at an altitude range of 3200 to 3700 m above sea level [27].

### 2.2. Climate, soil, and vegetation

GCCA is characterized by its plateau which cross-cut by various gorges and river valleys that flow west and eastwards. It straddles the catchment basins of two major rivers in East Africa–the Abay (the Blue Nile) and the Awash, and thus its conservation is critical to the health and functioning of those headwaters [41]. The swamps of the area increase water holding capacity and also limit runoff in the rainy seasons, thus providing flow all year round. Generally the soil of the Guassa area is deep and humic. However, on higher ground, the soil is shallow, and highly mineralized [28]. The climate in the guassa area varies widely due to the differences in elevation and the size of the mountain block. The annual mean rain fall is 828 mm. Temperatures are characterized by mild days and cold nights. In the drier months (December to February), daytime temperatures can rise to 25°C, while night time temperatures can drop to -7°C [40, 42]. The area gets its name from the "guassa grass" *Festuca macrophylla*, which is highly valued by the local community.

GCCA is a biodiversity hotspot, containing several endemic and threatened species [43]. The area is rich in biodiversity and contains several endemic and threatened species of flora and fauna species [28]. The vegetation of the guassa area is characterized by high Afroalpine Afroalpine vegetation with different plant community types, namely: *Euryops-Alchemilla* shrubland, *Festuca* grassland, *Euryops-Festuca* grassland, *Helichrysum—Festuca* grassland, Swamp Grassland and Erica moorland [42, 44]. GCCA is a place for important and endemic plant species including guassa grass (*F*. *macrophylla*), giant Lobelia (*Lobelia rhynchopetalum*), *Kniphofia foliosa* and *Alchemilla* species [28, 43]. There are about nine (which accounts to 23% mammal fauna of the country) endemic fauna including the Ethiopian wolf (*Canis simensis)*, gelada baboon (*Theropethicus gelada)* and Ethiopian Highland hare or Abyssinian hare (*Lepus starcki)* and about 114 (12% of 861 bird species) have been recorded in the GCCA [28]. The Guassa area is also home to the world’s largest concentration of the globally endangered Ankober serin (*Serinus ankoberensis)* and the spot-breasted plover (*Vannellus melocephalus*) [27].

### 2.3. Population and livelihood

The human population is predominantly of Amhara ethnicity and Orthodox Christian religion. The main economic activity of the people is farming which involves mainly crop production and livestock husbandry. The community can generate income from off-farm activities such as petty trading, selling local beer (‘Tella’), liquor (‘Arekie’) and local green tea. Wool processing (known in sheep and cattle rearing) is the most common off-farm activity of the Guassa community. The area is regarded as food insecure, and in many years, parts of the population depend on the national food aid program called safety-net [27].

The Guassa area is currently managed by nine farmers’ associations, local institutions that were created throughout Ethiopia in 1975 for rural administration. The community protects the area by promulgating various regulations that restrict the use of natural resources by the communities [28].

### 2.4. Soil sampling

First, we stratified the vegetation by type and purposefully selected eight blocks based on elevation (<3350 m.a.s.l/lower and >3350 m.a.s.l/upper elevation) and aspect. Each block has twenty 5m x 5m established plots, thus we have a total of 160 plots. We also categorized aspects in to four as NE, NW, SE and SW to compare the seed density in the soil for the target species along elevation gradient. The samples were taken from five subplots (1m x 1m each) (one at the center and the other four at the corners) to collect composite soil samples from the three separate layers. Within the 1m x 1m subplots, five subplots (5cm x 5cm) were established to take the soil seed bank sample. 225cm^3^ of soil seed bank samples were collected from each subplot using a digger. A total of 480 soil samples (3 vertically successive layers x 160 sample plots) were collected from the three separate soil layers. Each layer had 3 cm depth (0–3 cm, 3–6 cm and 6–9 cm) following [45–47]. The litter layer was deliberately removed. This is because, the probability of predation, rain-washing seeds from the surface resulted in poorly guided to conclude restoration potential of woody species from litter layer. To capture spatial heterogeneity of seed distribution [48, 49], soil samples from similar layers of the five subplots were mixed and put in one plastic bag to form composites samples and to reduce variability within the plot. The composite sample for each soil layer was again divided into five equal parts among which one (400 gm) was randomly selected for greenhouse germination.

During data collection, permission to collect samples from the study area was granted by the Guassa Community Conservation Area committee, which consists of nine individual representatives from each kebele and the governing body of the area. The Guassa Community Conservation Area is owned by the government and the local communities surrounding the area are formally the responsible body to manage and protect the conservation area. Moreover, there were no protected species sampled during this work. Sampling was completed within two weeks (February 13–30, 2021) to avoid differences between habitats, and thus any temporal bias in seed availability and composition following the method used by [50]. These samples were packed in plastic bags and transported to Addis Ababa University for greenhouse germination. The soil samples were first sieved using a mesh size of 2 mm to recover seeds of the different plant species [51]. The sieved soil samples were spread immediately in plastic trays in Addis Ababa University greenhouse for germination of the seeds. Each plastic tray (20cm x 3cm) was perforated at the bottom and plugged by cotton to facilitate proper drainage of water without losing soil. The seedling trays were kept continuously moist by daily watering following the method used by [50]. The emerging seedlings were identified, counted and recorded. Species identification was done using local reference material (Flora of Ethiopia and Eritrea Volumes [52–56]. Each of the plant specimens were pressed and deposited in the National Herbarium Ethiopia (ETH), at Addis Ababa University.

### 2.5. Data analysis techniques

All the collected data were organized in excel and checked for errors, normal distribution and homogeneity of variance before the data analysis were run [57, 58]. Using SAS statistical software version 9.0.1 (SAS Institute, 2001), all the collected data was fitted into a general linear model (GLM) and were analyzed using two-way ANOVA (Analysis of variance) to determine the abundance of *Festuca macrophylla* and *Helichrysum splendidum* in the soil seed bank along altitudinal gradient. Shannon diversity index was used to determine species diversity in the soil seedbank. Fisher’s least significant difference (LSD) test was employed to separate the means when significant F values were found at a significance level of p≤ 0.05. We calculated species abundance by totaling the number of seedlings that emerged from each sample. The composition, density and frequency of seeds in the soil were determined from the germination data recorded in the greenhouse experiment. The density of seeds was derived from the total number of seeds recovered from the soil samples.

Density is more useful in estimating the importance of a species. The sum of individuals per species is calculated in terms of species density per sampled area unit, such as hectare [57, 59].


$$D=\frac{The\;number\;of\;stems\;of\;a\;species\;}{Sampled\;area\;in\;hectar\;}\times 100$$

In order to analyze the diversity of soil seed bank, Shannon-Wiener diversity index (H′) was used following the method used by [60]. The Shannon index, which combines species richness with relative abundance, is widely used in species diversity studies [61]. The Shannon index expresses the relative evenness or equitability of species, gives weight to dominant species [62]. Total species diversity, richness and evenness of the soil seed bank of the study area were calculated taking the pooled seeds from the plots and the three soil layers found in each blocks.

The values of the Shannon-wiener diversity index usually falls between 1.5 and 3.5, although in exceptional cases, the value can exceed 4.5. The value of evenness index falls between zero and one. The higher the value of evenness index, the more even the species is in their distribution within the given area.

## 3. Results

### 3.1 Species composition of soil seed banks

**Fig 2:**
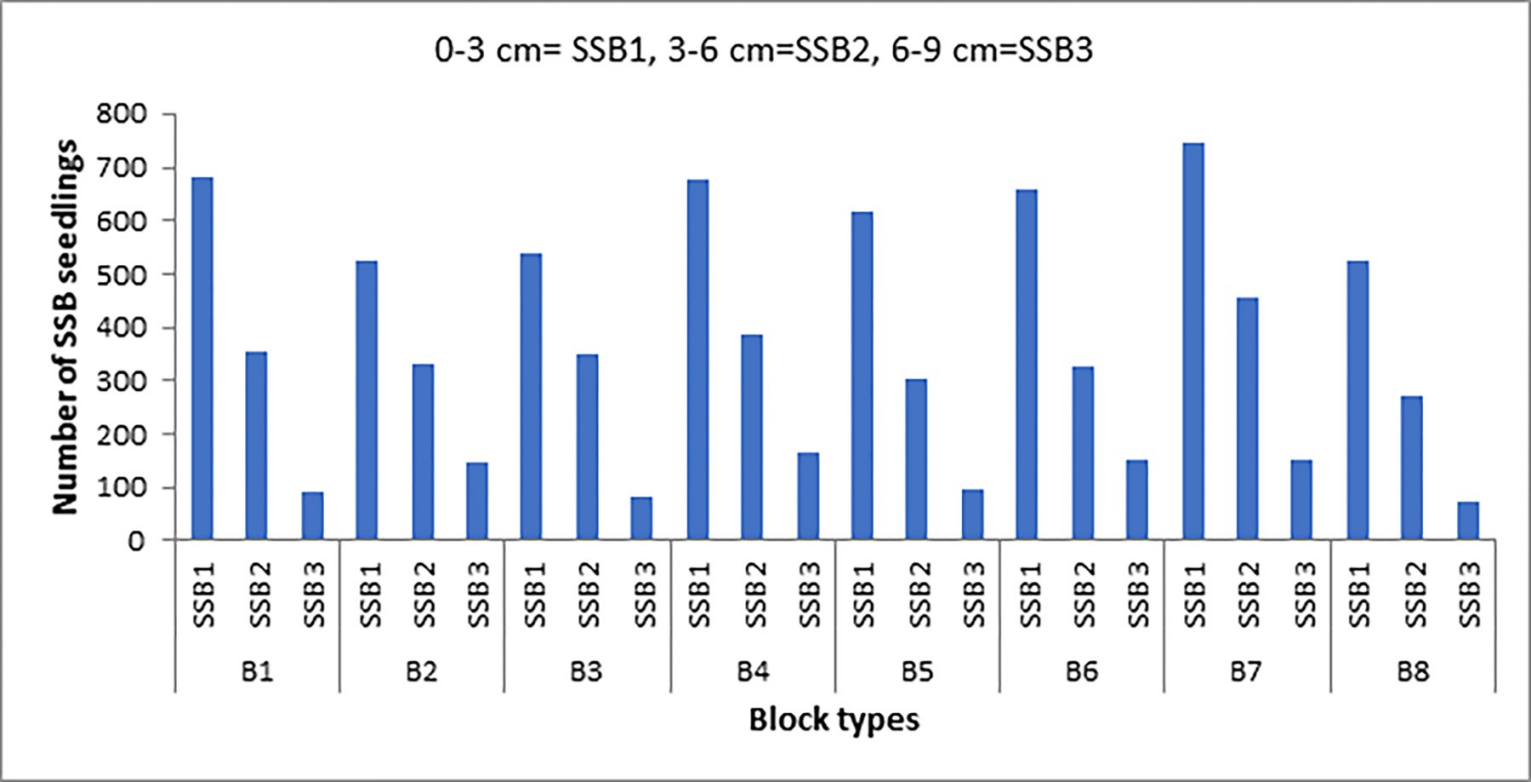
Number of seedlings recorded from soil seed banks of the upper 9 cm soil depth collected from GCCA. Key: SSB = soil seed bank, SSB1 = 0–3 cm, SSB2 = 3–6 cm, SSB3 = 6–9 cm.

The total number of species recorded was 74 species, representing 55 genera and 23 families from soil samples collected. Of the identified species recorded from the seed bank, 64 (86.49%) were herbs and 10 (13.51%) were shrub plants. Families with the highest number of species were Asteraceae (18 species), Poaceae (9 species), Scrophulariaceae (6 species), Cyperaceae (5 species), and the remaining families were represented by less than four species each. Five species namely: *Thymus schimperi* (Lamiaceae, 1457 seedlings), *H*. *splendidum (*Asteraceae, 1136 seedlings), *Trifolium polystachyum* (Fabaceae, 843 seedlings), *Trifolium usambarense* (Fabaceae, 752 seedlings) and *Euryops pinifolius* (Asteraceae, 701 seedlings) accounted for more than 56.3% of these seedlings (Table 1). From the three soil layers (i.e. 0–3 cm, 3–6 cm, 6–9 cm), high number of seedlings were recorded from soil seed bank of the upper layer (0–3 cm) (Fig 2).

**Table 1:** Top ranked species with their total number of seedlings in the GCCA.

Species	Family	Number of seedlings	Density (%)	Rank
*Thymus schimperi* Ronniger	Lamiaceae	1457	13.2	1
*Helichrysum splendidum* (Thumb.) Less	Asteraceae	1136	10.28	2
*Trifolium polystachyum* Fresen.	Fabaceae	843	7.63	3
*Trifolium usambarense* Taub.	Fabaceae	752	6.78	4
*Euryops pinifolius* A. Rich.	Asteraceae	701	6.32	5
*Alchemilla ellenbecki* Engl.	Rosaceae	610	5.49	6
*Alchemilla abyssinica* Fresen.	Rosaceae	577	5.19	7
*Festuca macrophylla* Hochst. ex A. Rich.	Poaceae	357	3.21	8
*Urtica simensis* Steudel	Urticaceae	336	3.02	9
*Cyperus elegantulus* Steud.	Cyperaceae	172	1.54	10

Eleven of the taxa are endemic to Ethiopia. These are: *Anchusa affinis*, *Cineraria abyssinica*, *Cynoglossum coerulem*, *Euryops pinifolius*, *F*. *macrophylla*, *kalanchoe petitiana*, *Kniphofia foliosa*, *Lobelia rhynchopetalum*, *Senecio schultzii*, *Senecio steudelii*, and *Urtica simensis*. In addition, there are also three near-endemic species to Ethiopia which are also found in Eritrea. These are: *Anchusa affinis*, *Plectocephalus varians* and *Thymus schimperi*. From these endemic taxa some of them like *F*. *macrophylla*, *Kniphofia foliosa*, *Urtica simensis* and *Cynoglossum coerulem* are stated in the IUCN Red list categories as least concern species.

### 3.2 Density of seeds in the soil

The maximum seed density was recorded in the first sampling layer (0–3 cm). Seed density and total species abundance decreases as the soil depth increases in the GCCA. The species with the highest soil seed densities in descending order include *Thymus schimperi*, *Helichrysum splendidum*, *Trifolium polystachyum*, *Trifolium usambarense*, *Euryops pinifolius*, and *Alchemilla ellenbecki* (Table 1).

### 3.3 Species richness, diversity and evenness of soil seed bank

The Shannon-Wiener diversity index for the diversity of soil seed bank at the study area generally demonstrated high values for the diversity of soil seed bank as it is greater than 2.5 (Table 2). The Shannon evenness index (E) had no consistence value among the soil layers in the study area; but totally viewed as 0–3 cm>6–9 cm>3–6 cm. Species in the middle layer (3–6 cm) are more evenly distributed than the others whereas the species in the bottom layer (6–9 cm) are less evenly distributed. Generally species richness decreased down the soil layers (Table 2).

**Table 2:** Soil seed bank species richness, diversity and evenness of GCCA.

Soil layers	Species richness (S)	Diversity Index (H’)	Shannon’s-Evenness (E)
0–3 cm	70	2.85	0.67
3–6 cm	53	3.05	0.72
6–9 cm	31	3.81	0.37

### 3.4 Vertical distribution of seeds in the soil seed bank

The depth distribution of the seed bank was consistent with the highest seed densities in the upper three centimeters (= 0–3 cm depth) of soil and a gradually decreasing number of species and densities of seeds with increasing soil depth. The soil seed bank density exhibited a declining trend with increasing soil depth accounting for 4934 seeds m^-2^ (0–3 cm), 2783 seeds m^-2^ (3–6 cm) and 949 seeds m^-2^ (6–9 cm). There was variation in relation to depth distribution that decreased to the lower centimeters for the sampled species (Table 3). Most of the species recorded in the soil seed banks had seeds in all the three different soil layers. Alternatively, there was variation in the depth distribution of seeds among different plant species. For instance, seeds of some species were recovered only from the upper three centimeters soil layers e.g. *Nepeta azurea*. On the other hand, most of the species have seeds which are distributed in all soil layers, e.g. *Alchemilla abyssinica*, *Alchemilla ellenbecki* and *Euryops pinifolius* (Table 3).

**Table 3:** Ten purposively selected plant species with their variation in relation to depth distribution of seeds from GCCA.

Layers	Density of seeds m^-2^										
	AA	AE	NA	EP		FM	HS	TS	TP	TU	US
0–3 cm	335	293	2		442	169	681	923	413	457	210
3–6 cm	234	205	0		218	134	263	408	272	208	99
6–9 cm	3	112	0		41	47	192	126	158	87	27
**Total**	572	610	2		701	350	1136	1457	843	752	336

(Abbreviations: AA = *Alchemilla abyssinica;* AE = *Alchemilla ellenbecki*; *NA = Nepeta azurea;* EP *Euryops pinifolius;* FM = *F*. *macrophylla;* HS = *H*. *splendidum;* TS = *Thymus schimperi;* TP = *Trifolium polystachyum;* TU = *Trifolium usambarense;* US = *Urtica simensis*).

### 3.5 Soil seed bank density of *Festuca macrophylla* and *Helichrysum splendidum*

**Fig 3:**
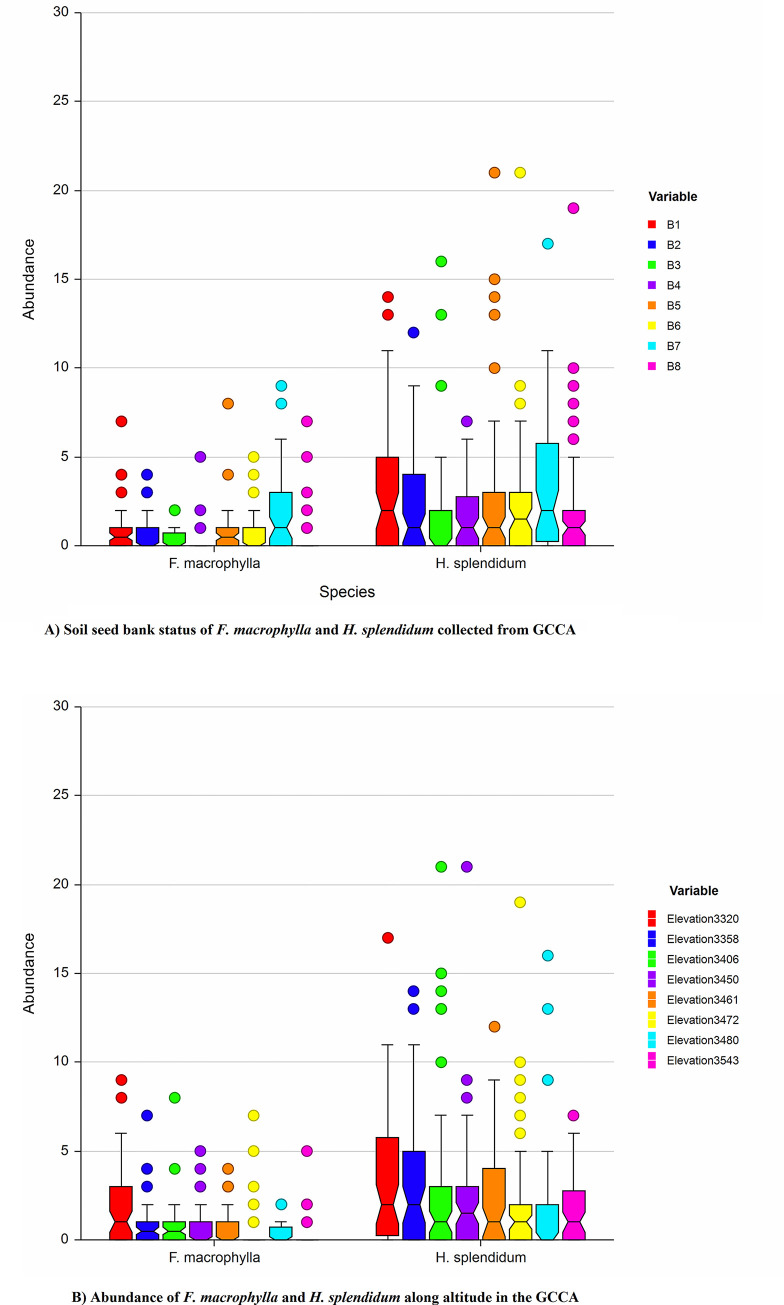
A) Soil seed bank status of *F*. *macrophylla* and *H*. *splendidum* collected from GCCA, B) Abundance of *F*. *macrophylla* and *H*. *splendidum* along altitude in the GCCA.

From the analysis of soil seed bank, the density of *H*. *splendidum* was found to be higher than seeds of *F*.*macrophylla* in each block both along aspect and altitudinal gradients (Fig 3A).

#### 3.5.1 Abundance of the seeds of *Festuca macrophylla* and *Helichrysum splendidum* plant species in relation to altitude and soil depth

The abundance of the seeds of the two target species (*F*. *macrophylla* and *H*. *splendidum*) in the soil seed bank showed significant differences. The pooled abundance and altitude were negatively correlated (Two-way ANOVA: P<0.05), where abundance of the two species showed decreasing pattern as altitude increase in the sample plots (Fig 3B).

Altitudinal difference had great impact on diversity and abundance of species in the study site. The lower altitude had a significant variation index compared to upper altitude gradient (Table 4).

**Table 4:** Interaction effect of altitude, aspect and soil depth on soil seed bank abundance of *F*. *macrophylla* and *H*. *splendidum*.

Lower elevation			
Treatment	*F*. *macrophylla* abundance		
Aspect*soil depth			
**Aspect**	**Soil depth**		
	0–3 (cm)	3–6 (cm)	6–9 (cm)
NW	18.33^bc^	5.00^d^	4.33^d^
NE	50.67^a^	24.00^b^	10.00^cd^
**P. value**	**0.0088**		
**Treatment**	***H*. *splendidum* abundance**		
Aspect*soil depth			
**Aspect**	**Soil depth**		
	0–3 (cm)	3–6 (cm)	6–9 (cm)
NW	110.33^a^	45.67^c^	36.00^cd^
NE	86.00^b^	33.33^d^	21.33^e^
**p. value**	**0.005**		
**Upper elevation**			
**Treatment**	***F*. *macrophylla* abundance**		
Aspect*soil depth			
**Aspect**	**Soil depth**		
	**0–3 (cm)**	**3–6 (cm)**	**6–9 (cm)**
**SW**	22.67^a^	19.00^ab^	14.33^bc^
**SE**	20.00^a^	13.00^c^	9.00^c^
**P. value**	**0.0052**		
**Treatment**	***H*. *splendidum* abundance**		
Aspect*soil depth			
**Aspect**	**Soil depth**		
	**0–3 (cm)**	**3–6 (cm)**	**6–9 (cm)**
**SW**	69.67^a^	40.00^c^	25.33^d^
**SE**	51.33^b^	22.67^d^	14.67^d^
**p. value**	**0.0051**		

Mean values followed by the same letters in each column and treatment showed no significant difference by LSD (p = 0.05). **KEY**: NW = North-West, NE = North-East, SW = South-West, SE = South-East

#### 3.5.2. Effect of aspect on the abundance of *F*. *macrophylla* and *H*. *splendidum* plant species

From the analysis done using SAS software, aspect significantly affected abundance of the two species, where both species with high abundance recorded higher at North-West and North-East direction followed by South-West and South-East direction (Table 5).

**Table 5:** Effect of aspect on *F*. *macrophylla* and *H*. *splendidum* abundance on lower and upper elevation of GCCA.

Lower elevation		
Treatment	*F*. *macrophylla* abundance	*H*. *splendidum* abundance
**Aspect**		
NW	28.22^a^	64.00^a^
NE	17.22^b^	46.89^b^
**P. value**	**< .0001**	**0.0001**
**Soil depth**		
0–3 cm	34.50^a^	98.17^a^
3–6 cm	14.50^b^	39.50^b^
6–9 cm	7.17^b^	28.67^c^
**p. value**	**<0.0001**	**<0.0001**
**Upper elevation**		
**Treatment**	***F*. *macrophylla* abundance**	***H*. *splendidum* abundance**
**Aspect**		
SW	9.67^a^	45.00^a^
SE	7.00^b^	29.56^b^
**P. value**	**0.0076**	**0.0002**
**Soil depth**		
0–3 cm	21.33^a^	60.50^a^
3–6 cm	16.00^b^	31.33^b^
6–9 cm	11.67^c^	20.00^c^
**p. value**	**0.0008**	**< .0001**

Mean values followed by the same letters in each column and treatment showed no significant difference by LSD (p = 0.05). **KEY**: NW = North-West, NE = North-East, SW = South-West, SE = South-East

#### 3.5.3. Depth distribution of *Festuca macrophylla* and *Helichrysum splendidum* plant species in the seed banks

Vertical distribution of soil seed bank of both *F*. *macrophylla* and *H*. *splendidum* showed similar patterns. The highest amount of seed was found within the first soil layer (0–3 cm) depth, while small amount for these species were found in the bottom layers (3–6 cm and 6–9 cm). It showed decreasing trend vertically following with increasing soil depth (Tables 4 and 5).

## 4. Discussion

Densities of seeds in the samples collected from the different plots varied greatly within and between the eight blocks as well as in the three different soil layers. The species composition and density of the soil seed banks in the study area depends on the type of plant species that existed in the conservation area. The findings of the soil seed bank study revealed that there are large quantities of seeds of herbaceous species in the soil. The predominance of herbaceous may be attributed to small seed size, which makes them more easily incorporated into the soil to form seed bank and also less prone to predation [63–65]. The endemic species account for about 14.86% of the total species, which is higher than the 10% endemism of the Ethiopian Flora. This implies that the area is rich in endemic species of plants. The Shannon diversity indices for the diversity of soil seed bank showed that species richness declined with altitude and along the depth. The study agrees with previous findings of unimodal relationships between altitude and plant species richness [66, 67]. Such relationships have been suggested as the consequence of productivity associated competitive exclusions at lower altitudes [68] and productivity-related dilutions of regional species pools at higher altitudes [69]. Despite the observed declines in species richness, no species evenness pattern were observed, so that not one species appears to have become dominant as species communities became diluted at higher altitudes. Although this result partly could have been caused by a declining species pool at higher altitudes [70], our observations support suggestions of strong abiotic regulation of plant diversity at higher altitudes [71], coupled with relaxed competition [67]. This variation can also be attributed to nature of the soil, slope, difference in magnitude and intensity of disturbances could add up to the variation in diversity and evenness of soil seed bank [72].

The present study revealed that *H*. *splendidum* had higher density of seeds in the soil than *F*. *macrophylla*. The possible reason for the existence of high seed of *H*. *splendidum* in the soil may be due to high seed production which may persist in the soil for a long period of time [73]. In addition, the species may have benefited from the current management system that forbid the local community from harvesting the shrub for fire and for other purposes and therefore, enabling the continuous production of seeds.

On the other hand, low seed density of *F*. *macrophylla* in the soil could be attributed to the light small sized seed and frequently found on the steep slope. These small sized seeds are mostly found on the steep slopes getting a chance to be washed by water [74]. Moreover, *F*. *macrophylla* is perennial herb and produce its seed every two year and grass harvesting or cutting (disturbance by the local community may be done before it produce seed [34–36] which may also result in low persistence seed in the soil [32, 75]. The current management system in GCCA that involves two-three years cycle of harvesting festuca grass for various uses may have led to decreasing seed production and thus less seed rain as the compared to other species. The dominance of the grass is possibly due to vegetative reproduction instead of germination from soil seedbank.

Patterns of abundance of *F*. *macrophylla* and *H*. *splendidum* were determined against altitude and aspect. The result indicated that abundance of *F*. *macrophylla* and *H*. *splendidum* negatively correlated with altitude (P<0.05). This result was similar with the one [76] who found that altitude is the most significant factor that affects species abundance and distribution in Afroalpine region. A reduction in species abundance might be due to harsh climatic condition and less competition as altitude increase [76]. According to [67] as altitude increases, richness of vegetation declines as a result of harsh climatic condition in the upper altitude caused by restriction in species’ expansion. Altitude was found to be very important in many studies in influencing the plant species distribution [77, 78]. Altitude was the main variable that determines the vertical distribution of vegetation whereas the horizontal distribution was affected by aspect [77].

Species abundance is also significantly affected by aspect. High number of *F*. *macrophylla* and *H*. *splendidum* recorded along North-West and North-East direction followed by South-West and South-East directions. The high number recorded in these directions may be attributed to the fact that seed dispersed by wind and also facilitated by human activity giving an opportunity to widely disperse in the soil [79]. But considering study area as a whole a high number of *H*. *splendidum* was possibility due to high disturbance pressure as a result of high population density living in the surrounding areas as compared to the other two aspects. In addition, high disturbance may favor increase in non-native species hence opened area give an opportunity to germinate seed in soil [44].

Vertical distribution of seeds of a species is assumed to reflect the longevity of its seeds in the soil [80]. The vertical distribution of the seeds in the soil showed that most habitat types have highest seed densities in the upper three centimeters of soil and gradually decreasing densities with increasing depth. The vertical distribution of the seeds of the two target species also showed similar trend [81–85]. The variations of seed density in successive layers may indicate *H*. *splendidum* had better seed longevity in the soil than *F*. *macrophylla* in addition to mode of seed dispersal and seed predation. It also suggests that if the upper soil layer is degraded by soil erosion or other factors, there may be variation in soil seed bank down to 9 cm.

The finding provides further evidences that *H*. *splendidum* showed significant increase in GCCA. The increase in *H*. *splendidum* cover confirms the problem of *H*. *splendidum* encroachment in the GCCA. This also confers with other study in the area. For example, the high-rate expansion of *H*. *splendidum* and its encroachment over other habitats, like *Festuca* grassland was pointed [30]. The *H*. *splendidum* free areas would be subject to competition for various uses such as agriculture land and grazing. The previous study indicated the expansion of farming at the western edge of GCCA [27]. Generally, *H*. *splendidum* increment was the predominant factor for the decrease in *F*. *macrophylla* and causes a decline in the status of the grassland in the study area [30]. Commonly *H*. *splendidum* encroachment was one but other drivers also listed as cause for the decreased of grass land cover. Much of the Ethiopian landscape from sea level up to 4,000 m is altered by agricultural activities, deforestation and overgrazing in order to fit the basic needs of a growing human population [86, 87].

## 5. Conclusions

Guassa Community Conservation Area is one of the protected areas in Ethiopia where the local community harvest guassa grass for different uses and other shrubs for firewood. The result of assessment of soil seed bank of GCCA revealed that the species recorded from soil seed bank were high. A total of 74 plant species were identified from which eleven taxa are endemic and three are near endemic to Ethiopia. The soil seed bank was dominated by the herbaceous flora and in terms of seed bank distribution, the upper 0–3 cm soil has high number of species when compared with the lower 3–6 cm and 6–9 cm soil. The study showed that abundance of the two target species was significantly affected by altitude and aspect. In addition, the current management system seems to favor the expansion of *H*. *splendidum*.

Evidence from this study showed that there is a significant increase in the expansion of *H*. *splendidum* encroachment in GCCA. The aboveground result also revealed and complemented that the density of *H*. *splendidum* is highly abundant than *F*. *macrophylla*. Thus, the GCCA has experiencing encroachment by *H*. *splendidum*. This study also concludes that the rate of the expansion of *H*. *splendidum* encroachment might risk the loss of other habitats (such as *Festuca grass land*, *Erica* and *other shrub lands)*. Furthermore, since the interest of local community to protect the GCCA is because of the interest on the *Festuca grassland*, then if the rate of *H*. *splendidum* encroachment over the *Festuca grassland* continuous, it might result a failure of interest by the local community to protect the GCCA. Thus, expansion of *H*. *splendidum* might result in the loss of *F*. *macrophylla*.

Finally, loss of such ecologically and economically valuable plant species would have great implications for the environment, biodiversity and socio-economic development of the communities. Therefore, conservation and sustainable utilization of *F*. *macrophylla* through different management approach such as reintroducing the “QERO” management system that has been exercised for centuries by the local community would bring a better conservation outcome especially when complimented with scientific management approach.

## Supporting information

S1 File All data generated and analyzed during this study are available as supplementary materials. (DOCX)

## Abbreviations

GCCA: Guassa Community Conservation Area
NPP: Net primary productivity
NE: North-East
NPP: Net primary productivity
NW: North-West
SE: South-East
SSB: Soil seed bank
SW: South-West

## References

[1] Kindu M, Schneider T, Teketay D, Knoke T. Land use/land cover change analysis using object-based classification approach in Munessa-Shashemene landscape of the Ethiopian highlands. Remote sensing. 2013 May 15;5(5):2411–35.

[2] Gashaw T, Dinkayoh T. Land use/land cover dynamics in Hulet Wogedamea Kebele, northern Ethiopia. Current Research in Agricultural Sciences. 2015;2(1):36–41.

[3] Tegene B. Land-cover/land-use changes in the derekolli catchment of the South Welo Zone of Amhara Region, Ethiopia. Eastern Africa Social Science Research Review. 2002;18(1):1–20.

[4] Bewket W. Land cover dynamics since the 1950s in Chemoga watershed, Blue Nile basin, Ethiopia. Mountain research and development. 2002 Aug;22(3):263–9.

[5] Tefera MM. Land-use/land-cover dynamics in Nonno district, central Ethiopia. Journal of Sustainable development in Africa. 2011;13(1):123–41.

[6] Molla MB. Land use/land cover dynamics in the central rift valley region of Ethiopia: Case of Arsi Negele District. African Journal of Agricultural Research. 2015 Jan 29;10(5):434–49.

[7] Alemu B, Garedew E, Eshetu Z, Kassa H. Land use and land cover changes and associated driving forces in north western lowlands of Ethiopia. International research journal of agricultural science and soil science. 2015 Jan;5(1):28–44.

[8] Bekele M. Forest property rights, the role of the state, and institutional exigency: the Ethiopian experience, (2003): 0073–0073.

[9] Eshetu Z, Högberg P. Reconstruction of forest site history in Ethiopian highlands based on 13C natural abundance of soils. AMBIO: A Journal of the Human Environment. 2000 Mar;29(2):83–9.

[10] Archer S, Schimel DS, Holland EA. Mechanisms of shrubland expansion: land use, climate or CO2?. Climatic change. 1995 Jan;29(1):91–9.

[11] Fang J, Chen A, Peng C, Zhao S, Ci L. Changes in forest biomass carbon storage in China between 1949 and 1998. Science. 2001 Jun 22;292(5525):2320–2. doi: 10.1126/science.1058629 11423660

[12] Van Auken OW. Shrub invasions of North American semiarid grasslands. Annual review of ecology and systematics. 2000 Nov;31(1):197–215.

[13] Maestre FT, Bowker MA, Puche MD, Belén Hinojosa M, Martínez I, García‐Palacios P, et al. Shrub encroachment can reverse desertification in semi‐arid Mediterranean grasslands. Ecology letters. 2009 Sep;12(9):930–41. doi: 10.1111/j.1461-0248.2009.01352.x 19638041

[14] Briggs JM, Knapp AK, Blair JM, Heisler JL, Hoch GA, Lett MS, et al. An ecosystem in transition: causes and consequences of the conversion of mesic grassland to shrubland. BioScience. 2005 Mar 1;55(3):243–54.

[15] Montané F, Casals P, Taull M, Lambert B, Dale MR. Spatial patterns of shrub cover after different fire disturbances in the Pyrenees. Annals of Forest Science. 2009 Sep 1;66(6):1–8.

[16] Lett MS, Knapp AK. Woody plant encroachment and removal in mesic grassland: production and composition responses of herbaceous vegetation. The American Midland Naturalist. 2005 Apr;153(2):217–31.

[17] Montane F, Romanya J, Rovira P, Casals P. Aboveground litter quality changes may drive soil organic carbon increase after shrub encroachment into mountain grasslands. Plant and Soil. 2010 Dec;337:151–65.

[18] Lett MS, Knapp AK, Briggs JM, Blair JM. Influence of shrub encroachment on aboveground net primary productivity and carbon and nitrogen pools in a mesic grassland. Canadian Journal of Botany. 2004 Sep 1;82(9):1363–70.

[19] Jackson RB, Banner JL, Jobbágy EG, Pockman WT, Wall DH. Ecosystem carbon loss with woody plant invasion of grasslands. Nature. 2002 Aug 8;418(6898):623–6. doi: 10.1038/nature00910 12167857

[20] Knapp AK, Briggs JM, Collins SL, Archer SR, BRET‐HARTE MS, et al. Shrub encroachment in North American grasslands: shifts in growth form dominance rapidly alters control of ecosystem carbon inputs. Global change biology. 2008 Mar;14(3):615–23.

[21] Van Auken OW. Causes and consequences of woody plant encroachment into western North American grasslands. Journal of environmental management. 2009 Jul 1;90(10):2931–42. doi: 10.1016/j.jenvman.2009.04.023 19501450

[22] Augustine DJ, Mcnaughton SJ. Regulation of shrub dynamics by native browsing ungulates on East African rangeland. Journal of Applied Ecology. 2004 Feb;41(1):45–58.

[23] Eldridge DJ, Bowker MA, Maestre FT, Roger E, Reynolds JF, Whitford WG. Impacts of shrub encroachment on ecosystem structure and functioning: towards a global synthesis. Ecology letters. 2011 Jul;14(7):709–22. doi: 10.1111/j.1461-0248.2011.01630.x 21592276 PMC3563963

[24] Oba G, Post E, Syvertsen PO, Stenseth NC. Bush cover and range condition assessments in relation to landscape and grazing in southern Ethiopia. Landscape ecology. 2000 Aug;15:535–46.

[25] Angassa A, Oba G. Relating long-term rainfall variability to cattle population dynamics in communal rangelands and a government ranch in southern Ethiopia. Agricultural systems. 2007 Jun 1;94(3):715–25.

[26] Yusuf H, Treydte AC, Demissew S, Woldu Z. Assessment of woody species encroachment in the grasslands of Nechisar National Park, Ethiopia. African Journal of Ecology. 2011 Dec;49(4):397–409.

[27] Ashenafi ZT. *Common property resource management of an Afro-alpine habitat*: *supporting a population of the critically endangered Ethiopian wolf (Canis simensis)* (Doctoral dissertation, University of Kent at Canterbury).

[28] Ashenafi ZT, Leader-Williams N. Indigenous common property resource management in the Central Highlands of Ethiopia. Human Ecology. 2005 Aug;33:539–63.

[29] Skarpe C. Shrub layer dynamics under different herbivore densities in an arid savanna, Botswana. Journal of Applied Ecology. 1990 Dec 1:873–85.

[30] Sillero-Zubiri C, Macdonald D. Ethiopian wolf: status survey and conservation action plan.

[31] VAN DER VARK AG. Seed banks and the management and restoration of natural vegetation. Ecology of soil seed banks. 1989:329–61. In: Leck M.A., Parker V.T. and Simpson R.L. (eds.). Ecology of Soil Seed Banks. Academic Press, San Diego.

[32] Lemenih M, Teketay D. Changes in soil seed bank composition and density following deforestation and subsequent cultivation of a tropical dry Afromontane forest in Ethiopia. Tropical Ecology. 2006;47(1):1–2.

[33] Reubens B, Heyn M, Gebrehiwot K, Hermy M, Muys B. Persistent soil seed banks for natural rehabilitation of dry tropical forests in northern Ethiopia. Tropicultura. 2007;25(4):204.

[34] Steger C, Nigussie G, Alonzo M, Warkineh B, Van Den Hoek J, Fekadu M, et al. Knowledge coproduction improves understanding of environmental change in the Ethiopian highlands. Ecology and Society. 2020 Jan;25(2).

[35] Steger C, Gebrehiwot K, Chengere SA, Marinkovich J, Dullo BW, Zewde SW, et al. Mental models of a social-ecological system facilitate social learning among a diverse management team. Environmental Science & Policy. 2021 Aug 1;122:127–38.

[36] Steger C, Boone RB, Dullo BW, Evangelista P, Alemu S, Gebrehiwot K, et al. Collaborative agent-based modeling for managing shrub encroachment in an Afroalpine grassland. Journal of Environmental Management. 2022 Aug 15;316:115040. doi: 10.1016/j.jenvman.2022.115040 35594826

[37] Brooks TM, Mittermeier RA, Mittermeier CG, Da Fonseca GA, Rylands AB, Konstant WR, et al. Habitat loss and extinction in the hotspots of biodiversity. Conservation biology. 2002 Aug;16(4):909–23.

[38] Hoffman M, Koenig K, Bunting G, Costanza J, Williams KJ. Biodiversity hotspots (version 2016.1). Zenodo doi: 10.5281/zenodo.2016;3261807 Accessed: May 19, 2022.

[39] World Conservation Monitoring Centre Biodiversity Data Sourcebook. World Conservation Press, Cambridge, UK. 1994.

[40] Wube S, Lemessa D, Dullo BW. Factors driving the expansion of Helichrysum splendidum in Menz-Guassa community conservation area of the Afroalpine ecosystem of Ethiopia. East African Journal of Sciences. 2021 Jun 2;15(1):17–24.

[41] Moges E. *Population structure*, *behavioural ecology and habitat vulnerability of gelada (Theropithecus gelada) in Guassa Community Protected Area*, *central Ethiopia* (Doctoral dissertation, Addis Ababa University). 2015.

[42] Simeneh G. Habitat use and diet of golden jackal (Canis aureus) and human-carnivore conflict in Guassa community conservation area, Menz. Addis Ababa, Ethiopia: Addis Ababa University. 2010.

[43] Ashenafi ZT, Leader-Williams N, Coulson T. Consequences of human land use for an Afro-alpine ecological community in Ethiopia. Conservation and Society. 2012 Jan 1;10(3):209–16.

[44] Beyene H. *Population estimate and structure of the gelada baboon*, *Theropithecus gelada*, *in the guassa community conservation area*, *central Ethiopia* (Doctoral dissertation, M. Sc. thesis, Addis Ababa University, Addis Ababa). 2010.

[45] Price JN, Wright BR, Gross CL, Whalley WR. Comparison of seedling emergence and seed extraction techniques for estimating the composition of soil seed banks. Methods in Ecology and Evolution. 2010 Jun;1(2):151–7.

[46] Li Q, Fang H, Cai Q. Persistent soil seed banks along altitudinal gradients in the Qilian Mountains in China and their significance for conservation management. African Journal of Agricultural Research. 2011 May 18;6(10):2329–40.

[47] Wang J, Huang L, Ren H, Sun Z, Guo Q. Regenerative potential and functional composition of soil seed banks in remnant evergreen broad-leaved forests under urbanization in South China. Community Ecology. 2015 Jun;16(1):86–94.

[48] Niknam P, Erfanzadeh R, Ghelichnia H, Cerdà A. Spatial variation of soil seed bank under cushion plants in a subalpine degraded grassland. Land Degradation & Development. 2018 Jan;29(1):4–14.

[49] Erfanzadeh R, Shayesteh Palaye AA, Ghelichnia H. Shrub effects on germinable soil seed bank in overgrazed rangelands. Plant Ecology & Diversity. 2020 Mar 3;13(2):199–208.

[50] López-Toledo L, Martínez-Ramos M. The soil seed bank in abandoned tropical pastures: source of regeneration or invasion? Revista mexicana de biodiversidad. 2011 Jun;82(2):663–78.

[51] Daïnou K, Bauduin A, Bourland N, Gillet JF, Fétéké F, Doucet JL. Soil seed bank characteristics in Cameroonian rainforests and implications for post-logging forest recovery. Ecological Engineering. 2011 Oct 1;37(10):1499–506.

[52] Edwards S, Tadese M, Hedberg I. Flora of Ethiopia and Eritrea vol. 2, Part 2: canellaceae to euphorbiaceae. AAU; 1995.

[53] Edwards S, Demissew S, Hedberg I. Flora of Ethiopia and Eritrea: Hydrocharitaceae to Arecaceae. The National Herbarium, Addis Ababa University; 1997.

[54] Edwards S, Tadesse M, Demissew S, Hedberg I. Flora of Ethiopia and Eritrea. Magnoliaceae to Flacourtiaceae (vol. 2:1). Department of Systematic Botany, Uppsala, Sweden: The National Herbarium, Addis Ababa, Ethiopia. 2000, pp. 332.

[55] Hedberg I, Edwards S. Flora of Ethiopia and Eritrea. Pittosporaceae to Araliaceae. (vol. 3). Department of Systematic Botany, Uppsala, Sweden: The National Herbarium, Addis Ababa, Ethiopia. 1989, pp. 659.

[56] Hedberg I, Edwards S, Nemomissa S. Flora of Ethiopia and Eritrea Apiaceae to Dipsacaceae. (vol. 4:1). Department of Systematic Botany, Uppsala, Sweden: The National Herbarium, Addis Ababa, Ethiopia. 2003, pp. 352.

[57] Erfanzadeh R, Hazhir S, Jafari M. Effect of cushion plants on the soil seed bank in overgrazed semiarid regions. Land Degradation & Development. 2020 May 15;31(8):990–1000.

[58] Yusefi H, Erfanzadeh R, Esmailzadeh O. Effect of wild boar disturbances on the soil seed bank in alpine plant communities. Land Degradation & Development. 2023 Feb 28;34(4):1225–34.

[59] Mueller-Dombois D, Ellenberg H. Aims and methods of vegetation ecology. Wiley; 1974.

[60] Tesfaye G, Teketay D, Fetene M, Beck E. Regeneration of seven indigenous tree species in a dry Afromontane forest, southern Ethiopia. Flora-Morphology, Distribution, Functional Ecology of Plants. 2010 Jan 1;205(2):135–43.

[61] Kent M. Vegetation description and data analysis: a practical approach. John Wiley & Sons; 2011 Nov 14.

[62] Whittaker RH. Evolution and measurement of species diversity. Taxon. 1972 May;21(2–3):213–51.

[63] Thompson K. Seeds and seed banks. New phytologist. 1987 May;106:23–34.

[64] Guo Q, Rundel PW, Goodall DW. Horizontal and vertical distribution of desert seed banks: patterns, causes, and implications. Journal of arid environments. 1998 Mar 1;38(3):465–78.

[65] Luzuriaga AL, Escudero A, Olano JM, Loidi J. Regenerative role of seed banks following an intense soil disturbance. Acta Oecologica. 2005 Jan 1;27(1):57–66.

[66] Rahbek C. The elevational gradient of species richness: a uniform pattern?. Ecography. 1995 Jun 1:200–5.

[67] Bruun HH, Moen J, Virtanen R, Grytnes JA, Oksanen L, Angerbjörn A. Effects of altitude and topography on species richness of vascular plants, bryophytes and lichens in alpine communities. Journal of Vegetation Science. 2006 Feb;17(1):37–46.

[68] Rosenzweig ML. Paradox of enrichment: destabilization of exploitation ecosystems in ecological time. Science. 1971 Jan 29;171(3969):385–7. doi: 10.1126/science.171.3969.385 5538935

[69] Huston MA. Local processes and regional patterns: appropriate scales for understanding variation in the diversity of plants and animals. Oikos. 1999 Sep 1:393–401.

[70] Dalerum F, de Vries JL, Pirk CW, Cameron EZ. Spatial and temporal dimensions to the taxonomic diversity of arthropods in an arid grassland savannah. Journal of Arid Environments. 2017 Sep 1;144:21–30.

[71] Sang W. Plant diversity patterns and their relationships with soil and climatic factors along an altitudinal gradient in the middle Tianshan Mountain area, Xinjiang, China. Ecological Research. 2009 Mar;24:303–14.

[72] Kellerman MJ, Van Rooyen MW. Seasonal variation in soil seed bank size and species composition of selected habitat types in Maputaland, South Africa. Bothalia. 2007 Aug 18;37(2):249–58.

[73] Wang SM, Zhang X, Li Y, Zhang L, Xiong YC, Wang G. Spatial distribution patterns of the soil seed bank of Stipagrostis pennata (Trin.) de Winter in the Gurbantonggut Desert of north-west China. Journal of Arid Environments. 2005 Oct 1;63(1):203–22.

[74] Looney PB, Gibson DJ. The relationship between the soil seed bank and above‐ground vegetation of a coastal barrier island. Journal of Vegetation Science. 1995 Dec;6(6):825–36.

[75] Levassor C, Ortega M, Peco B. Seed bank dynamics of Mediterranean pastures subjected to mechanical disturbance. Journal of Vegetation Science. 1990 Jun;1(3):339–44.

[76] Melese GT, Tsegay BA, Kassa GM. Effects of environmental variables on the patterns of plant community distribution in the afro-alpine vegetation of simien mountains national park, ethiopia. Journal of Biotechnology International. 2017 Oct 7;10(1):8–21.

[77] Jin XM, Zhang YK, Schaepman ME, Clevers JG, Su Z, Cheng J, et al. Impact of elevation and aspect on the spatial distribution of vegetation in the Qilian mountain area with remote sensing data. International Society for Photogrammetry and Remote Sensing.

[78] KHALIK KA, El-Sheikh M, El-Aidarous A. Floristic diversity and vegetation analysis of wadi Al-Noman, Mecca, Saudi Arabia. Turkish Journal of Botany. 2013;37(5):894–907.

[79] Schimmel J, Granstrom A. Fire severity and vegetation response in the boreal Swedish forest. Ecology. 1996 Jul;77(5):1436–50.

[80] Teketay D, Granström A. Soil seed banks in dry Afromontane forests of Ethiopia. Journal of vegetation Science. 1995 Dec;6(6):777–86.

[81] Teketay D, Granström A. Germination ecology of forest species from the highlands of Ethiopia. Journal of tropical ecology. 1997 Nov;13(6):805–31.

[82] Argaw M, Teketay D, Olsson M. Soil seed flora, germination and regeneration pattern of woody species in an Acacia woodland of the Rift Valley in Ethiopia. Journal of Arid Environments. 1999 Dec 1;43(4):411–35.

[83] Tekle K, Bekele T. The role of soil seed banks in the rehabilitation of degraded hillslopes in Southern Wello, Ethiopia 1. Biotropica. 2000 Mar;32(1):23–32.

[84] Senbeta F, Teketay D. Regeneration of indigenous woody species under the canopies of tree plantations in Central Ethiopia. Tropical Ecology. 2001;42(2):175–85.

[85] Wassie A, Teketay D. Soil seed banks in church forests of northern Ethiopia: Implications for the conservation of woody plants. Flora-Morphology, Distribution, Functional Ecology of Plants. 2006 Jan 2;201(1):32–43.

[86] Hillman JC. Ethiopia: Compendium of wildlife conservation information. Ethiopian Wildlife Conservation Organisation; 1993.

[87] Williams VL, Witkowski ET, Balkwill K. The use of incidence-based species richness estimators, species accumulation curves and similarity measures to appraise ethnobotanical inventories from South Africa. Biodiversity and Conservation. 2007 Aug;16:2495–513.

