# Supplementary material for: Quantifying shrub encroachment through soil seed bank analysis in the Ethiopian highlands
Source: PLoS One. 2023 Aug 21;18(8):e0288804. doi: 10.1371/journal.pone.0288804 (PMC10441778; doi:10.1371/journal.pone.0288804)
Supplement: S1 File — (DOCX) [file pone.0288804.s001.docx]

S1 Table. List of soil seed bank species obtained via germination from GCCA.

| **S. No** | **Scientific name of species with authority name(s)** | **Family name** | **Habit** |
| --- | --- | --- | --- |
| 1 | *Aeonium leucoblepharum* A. Rich. | Crassulaceae | H |
| 2 | *Agrocharis melanantha* Hochst. | Apiaceae | H |
| 3 | *Agrostis gracilifolia* C. E. Hubb | Poaceae | H |
| 4 | *Agrostis quinqueseta* (Hochst. ex Steud.) Hochst. | Poaceae | H |
| 5 | *Alchemilla abyssinica* Fresen. | Rosaceae | H |
| 6 | *Alchemilla ellenbecki* Engl. | Rosaceae | H |
| 7 | *Alchemilla kiwuensis* Engl. | Rosaceae | H |
| 8 | *Anchusa affinis* R. Br. Ex DC. | Boraginaceae | H |
| 9 | *Andropogon lima* (Hack.) stapf | Poaceae | H |
| 10 | *Andropogon amethystinus* Steud. | Poaceae | H |
| 11 | *Anthemis tigreensis* J. Gay ex A. Rich. | Asteraceae | H |
| 12 | *Argyrolobium ramosissimum* Bak. | Fabaceae | H |
| 13 | *Argyrolobium rupestre* (E. Mey.) Walp. | Fabaceae | H |
| 14 | *Artemisia abyssinica* Sch. Bip. Ex A. Rich. | Asteraceae | H |
| 15 | *Bartsia londiflora* Hochst.ex Benth. | Scrophulariaceae | SH |
| 16 | *Carduus schimperi* Sch. Bip. Ex A. Rich. | Asteraceae | H |
| 17 | *Carex conferta* Hochst.ex Benth. | Cyperaceae | H |
| 18 | *Carex monostachya* A.Rich | Cyperaceae | H |
| 19 | *Cineraria abyssinica* Sch. Bip. Ex A. Rich. | Asteraceae | H |
| 20 | *Conyza pyropappa* Sch. Bip. Ex A. Rich. | Asteraceae | SH |
| 21 | *Conyza stricta* Willd | Asteraceae | H |
| 22 | *Crassula alba* Forssk. | Crasulaceae | H |
| 23 | *Cynoglosum amplifolium* Hochst.ex Benth. | Boraginaceae | H |
| 24 | *Cynoglosum coeruleum* Hochst.ex Benth. | Boraginaceae | H |
| 25 | *Cyperus elegantulus* Steud. | Cyperaceae | H |
| 26 | *Cyperus rigidifolius* Steud. | Cyperaceae | H |
| 27 | *Dicrocephala chrysanthemifolia* DC. | Asteraceae | H |
| 28 | *Dipsacus pinnatifidus* Steud. ex A. Rich. | Dipsacaceae | H |
| 29 | *Epilobium stereophyllum* Fresen. | Onagraceae | H |
| 30 | *Erica arborea* L. | Ericaceae | SH |
| 31 | *Erica tenuipilosa* (Engl. ex Alm & Fries) Cheek | Ericaceae | SH |
| 32 | *Erigeron alpines* L. | Asteraceae | H |
| 33 | *Euryops pinifolius* A. Rich. | Asteraceae | SH |
| 34 | *Festuca abyssinica* Hochst.ex Benth. | Poaceae | H |
| 35 | *Festuca macrophylla* Hochst.ex Benth. | Poaceae | H |
| 36 | *Festuca richardii* E.B. Alexeev | Poaceae | H |
| 37 | *Galium simense* Fresen. | Rubiaceae | H |
| 38 | *Hebenstretia angolensis* Rolfe. | Scrophulariaceae | SH |
| 39 | *Hedbergia abyssinica* (Hochst.ex Benth.) | Scrophulariaceae | H |
| 40 | *Helichrysum formosissimum* Sch. Bip. Ex A. Rich. | Asteraceae | H |
| 41 | *Helichrysum stenopterum* DC. | Asteraceae | H |
| 42 | *Helichrysum forsskahilii* (J.F. Gmel.) Hilliard & Burtt | Asteraceae | H |
| 43 | *Helichrysum splendidum* (Thumb.) Less | Asteraceae | SH |
| 44 | *Helictotrichon elongatum* (Hochst. ex. A. Rich.) C. E. Hubb. | Poaceae | H |
| 45 | *Hesperantha petitiana* (A. Rich.) Baker | Iridaceae | H |
| 46 | *Hypericum revolutum* Vahl | Hypericaceae | SH |
| 47 | *Isolepis costata* A. Rich. | Cyperaceae | H |
| 48 | *kalanchoe petitiana* A. Rich | Crassulaceae | SH |
| 49 | *Kniphofia foliosa* Hochst. | Asphodelaceae | H |
| 50 | *Lobelia rhynchopetalum* Hemsl. | Lobeliaceae | H |
| 51 | *Luzula abyssinica* Parl. | Juncaceae | H |
| 52 | *Nepeta azurea* R.Br. ex Benth. | Lamiaceae | H |
| 53 | *Pimpinella oreophila* Hook. | Apiaceae | H |
| 54 | *Plectocephalus varians* (A.Rich.) C. Jeffrey ex. Cufod. | Asteraceae | H |
| 55 | *Ranunculus multifidus* Forssk. | Ranunculaceae | H |
| 56 | *Rhabdotosperma scrophularifolia* (Hochst. ex A. Rich.) Hartle | Scrophulariaceae | H |
| 57 | *Rubus volkensii* Engl. | Rosaceae | SH |
| 58 | *Rumex abyssinicus* Jacq. | Polygonaceae | H |
| 59 | *Rumex nepalensis* Spreng. | Polygonaceae | H |
| 60 | *Rytidosperma subulata* (A. Rich.) Cope | Poaceae | H |
| 61 | *Salvia merjamie* Forssk. | Lamiaceae | H |
| 62 | *Satureja pseudosimensis* Brenan | Lamiaceae | H |
| 63 | *Scabiosa columbaria* L. | Dipsacaceae | H |
| 64 | *Senecio ragazi* Chiov. | Asteraceae | H |
| 65 | *Senecio schulzii* Hochst. ex. A. Rich. | Asteraceae | H |
| 66 | *Senecio steudelii* Sch. Bip. ex A. Rich. | Asteraceae | H |
| 67 | *Senecio subsessilis* Oliv. & Hiern | Asteraceae | H |
| 68 | *Swertia kilimandscharica* Engl. | Gentianaceae | H |
| 69 | *Thymus schimperi* Ronniger | Lamiaceae | H |
| 70 | *Trifolium polystachyum* Fresen. | Fabaceae | H |
| 71 | *Trifolium usambarense* Taub. | Fabaceae | H |
| 72 | *Urtica simensis* Steudel | Urticaceae | H |
| 73 | *Verbascum sinaiticum* Benth. | Scrophulariaceae | H |
| 74 | *Veronica glandulosa* Hochst .ex Benth. | Scrophulariaceae | H |

(Note: H = Herb, SH = Shrub)

S2 Table. List of plant families with number of genera, species and percentage obtained from GCCA.

| **Family** | **Genera** | **Species** | **Percentage** |
| --- | --- | --- | --- |
| Apiaceae | 2 | 2 | 2.7 |
| Asphodelaceae | 1 | 1 | 1.4 |
| Asteraceae | 12 | 18 | 24.3 |
| Boraginaceae | 2 | 3 | 4.1 |
| Crassulaceae | 3 | 3 | 4.1 |
| Cyperaceae | 3 | 5 | 6.8 |
| Dipsacaceae | 2 | 2 | 2.7 |
| Ericaceae | 1 | 2 | 2.7 |
| Fabaceae | 2 | 4 | 5.4 |
| Gentianaceae | 1 | 1 | 1.4 |
| Hypericaceae | 1 | 1 | 1.4 |
| Iridiaceae | 1 | 1 | 1.4 |
| Juncaceae | 1 | 1 | 1.4 |
| Lamiaceae | 4 | 4 | 5.4 |
| Lobeliaceae | 1 | 1 | 1.4 |
| Onagraceae | 1 | 1 | 1.4 |
| Poaceae | 5 | 9 | 12.2 |
| Polygonaceae | 1 | 2 | 2.7 |
| Ranunculaceae | 1 | 1 | 1.4 |
| Rosaceae | 2 | 4 | 5.4 |
| Rubiaceae | 1 | 1 | 1.4 |
| Scrophulariaceae | 6 | 6 | 8.1 |
| Urticaceae | 1 | 1 | 1.4 |

S3 Table. The densities of seeds (number of seeds m^-2^) recovered from the three different layers of the samples collected from GCCA.

| **S.No** | **SPECIES NAME** | **SOIL LAYERS** | | | |
| --- | --- | --- | --- | --- | --- |
|  |  | **0 – 3 cm** | **3-6 cm** | **6-9 cm** | **Total** |
| 1 | *Aeonium* *leucoblepharum* A. Rich. | 42 | 33 | 0 | 75 |
| 2 | *Agrocharis* *melanantha* Hochst. | 40 | 18 | 4 | 62 |
| 3 | *Agrostis* *gracilifolia* C. E. Hubb | 53 | 36 | 7 | 96 |
| 4 | *Agrostis* *quinqueseta* (Hochst. ex Steud.) Hochst. | 74 | 69 | 1 | 144 |
| 5 | *Alchemilla* *abyssinica* Fresen*.* | 335 | 234 | 3 | 572 |
| 6 | *Alchemilla* *ellenbecki* Engl*.* | 293 | 205 | 112 | 610 |
| 7 | *Alchemilla* *kiwuensis* Engl. | 85 | 57 | 1 | 143 |
| 8 | *Anchusa* *affinis* R.Br. ex DC. | 25 | 21 | 0 | 46 |
| 9 | *Andropogon* *lima* (Hack.) Stapf | 30 | 31 | 1 | 62 |
| 10 | *Andropogon* *amethystinus* Steud. | 35 | 24 | 1 | 60 |
| 11 | *Anthemis* *tigreensis* J. Gay ex A*.* Rich*.* | 64 | 53 | 11 | 128 |
| 12 | *Argyrolobium* *ramosissimum* Bak*.* | 40 | 30 | 1 | 71 |
| 13 | *Argyrolobium* *rupestre* (E. Mey.) Walp. | 14 | 14 | 4 | 32 |
| 14 | *Artemisia* *abyssinica* Sch. Bip. ex A*.* Rich. | 10 | 13 | 0 | 23 |
| 15 | *Bartsia* *longiflora* Hochst.ex Benth. | 1 | 2 | 2 | 5 |
| 16 | *Carduus* *schimperi* Sch. Bip. ex A. Rich. | 2 | 6 | 2 | 10 |
| 17 | *Carex* *conferta* Hochst*.* ex A. Rich | 1 | 3 | 1 | 5 |
| 18 | *Carex* *monostachya* A. Rich*.* | 15 | 12 | 2 | 29 |
| 19 | *Cineraria* *abyssinica* Sch. Bip. ex A. Rich. | 37 | 32 | 28 | 97 |
| 20 | *Conyza* *pyrrhopappa* Sch. Bip. ex A. Rich. | 10 | 11 | 0 | 21 |
| 21 | *Conyza* *stricta* Willd | 9 | 5 | 3 | 17 |
| 22 | *Crassula* *alba* Forssk. | 5 | 7 | 1 | 13 |
| 23 | *Cynoglosum* *amplifolium* Hochst. ex A. DC. | 18 | 21 | 1 | 40 |
| 24 | *Cynoglosum* *coeruleum* Hochst. ex A. DC. | 20 | 17 | 9 | 46 |
| 25 | *Cyperus* *elegantulus* Steud. | 99 | 49 | 19 | 167 |
| 26 | *Cyperus* *rigidifolius* Steud. | 81 | 51 | 11 | 143 |
| 27 | *Dicrocephala* *chrysanthemifolia* DC. | 4 | 5 | 2 | 11 |
| 28 | *Dipsacus pinnatifidus* Steud. ex A. Rich. | 3 | 15 | 6 | 24 |
| 29 | *Epilobium stereophyllum* Fresen. | 6 | 8 | 0 | 14 |
| 30 | *Erica arborea* L. | 11 | 2 | 7 | 20 |
| 31 | *Erica tenuipilosa* (Engl. ex Alm & Fries) Cheek | 1 | 0 | 0 | 1 |
| 32 | *Erigeron alpinus* L. | 4 | 4 | 2 | 10 |
| 33 | *Euryops pinifolius* A. Rich. | 442 | 218 | 41 | 701 |
| 34 | *Festuca abyssinica,* Hochst. ex A. Rich. | 31 | 14 | 1 | 46 |
| 35 | *Festuca macrophylla* Hochst. ex A. Rich. | 169 | 134 | 47 | 350 |
| 36 | *Festuca richardii* E.B.Alexeev | 0 | 1 | 0 | 1 |
| 37 | *Galium simense* Fresen. | 6 | 6 | 0 | 12 |
| 38 | *Hebenstretia angolensis* Rolfe. | 4 | 5 | 0 | 9 |
| 39 | *Hedbergia abyssinica* (Hochst. ex Benth. | 1 | 0 | 1 | 2 |
| 40 | *Helichrysum formosissimum* Sch. Bip. ex A. Rich. | 4 | 2 | 3 | 9 |
| 41 | *Helichrysum stenopterum* DC. | 9 | 6 | 3 | 18 |
| 42 | *Helichrysum forsskahlii* (J.F. Gmel.) Hilliard& Burtt | 8 | 5 | 4 | 17 |
| 43 | *Helichrysum splendidum* (Thumb.) Less | 681 | 263 | 192 | 1136 |
| 44 | *Helictotrichon elongatum* (Hochst. ex. A. Rich.) C. E. Hubb. | 5 | 4 | 2 | 11 |
| 45 | *Hesperantha petitiana* (A. Rich.) Baker | 4 | 3 | 3 | 10 |
| 46 | *Hypericum revolutum* Vahl | 25 | 14 | 3 | 42 |
| 47 | *Isolepis costata* A. Rich. | 7 | 2 | 3 | 12 |
| 48 | *kalanchoe petitiana* A. Rich. | 10 | 4 | 4 | 18 |
| 49 | *Kniphofia foliosa* Hochst. | 0 | 1 | 0 | 1 |
| 50 | *Lobelia rhynchopetalum* Hemsl. | 1 | 0 | 0 | 1 |
| 51 | *Luzula abyssinica* Parl. | 2 | 1 | 0 | 3 |
| 52 | *Nepeta azurea* R.Br. ex Benth. | 2 | 0 | 0 | 2 |
| 53 | *Pimpinella oreophila* Hook. | 1 | 1 | 1 | 3 |
| 54 | *Plectocephalus varians* (A.Rich.) C. Jeffrey ex. Cufod. | 1 | 2 | 0 | 3 |
| 55 | *Ranunculus multifidus* Forssk. | 4 | 2 | 0 | 6 |
| 56 | *Rhabdotosperma scrophularifolia* (Hochst. ex A. Rich.) Hartle | 2 | 2 | 0 | 4 |
| 57 | *Rubus volkensii* Engl. | 5 | 1 | 0 | 6 |
| 58 | *Rumex abyssinicus* Jacq. | 2 | 1 | 0 | 3 |
| 59 | *Rumex nepalensis* Spreng. | 1 | 1 | 0 | 2 |
| 60 | *Rytidosperma subulata* (A. Rich.) Cope | 2 | 1 | 0 | 3 |
| 61 | *Salvia merjamie* Forssk. | 4 | 1 | 0 | 5 |
| 62 | *Satureja pseudosimensis* Brenan | 2 | 1 | 0 | 3 |
| 63 | *Scabiosa columbaria* L. | 1 | 2 | 0 | 3 |
| 64 | *Senecio ragazi* Chiov. | 2 | 1 | 0 | 3 |
| 65 | *Senecio schulzii* Hochst. ex. A. Rich. | 2 | 1 | 0 | 3 |
| 66 | *Senecio steudelii* Sch. Bip. ex A. Rich. | 3 | 2 | 0 | 5 |
| 67 | *Senecio subsessilis* Oliv. & Hiern | 1 | 1 | 0 | 2 |
| 68 | *Swertia kilimandscharica* Engl. | 6 | 1 | 1 | 8 |
| 69 | *Thymus* *schimperi* Ronniger | 923 | 408 | 126 | 1457 |
| 70 | *Trifolium polystachyum* Fresen. | 413 | 272 | 158 | 843 |
| 71 | *Trifolium usambarense* Taub. | 457 | 208 | 87 | 752 |
| 72 | *Urtica simensis* Steudel | 210 | 99 | 27 | 336 |
| 73 | *Verbascum sinaiticum* Benth. | 7 | 3 | 0 | 10 |
| 74 | *Veronica glandulosa* Hochst .ex Benth. | 7 | 1 | 0 | 8 |
|  | *Total* |  |  |  | 8666 |

S4 Table. Density and frequency of species collected from GCCA.

| **S. No** | **Scientific name** | **Seedlings count** | **Number of plots in which spp. occur** | **Density** | **Frequency** |
| --- | --- | --- | --- | --- | --- |
| 1 | *Aeonium* *leucoblepharum* A. Rich. | 76 | 35 | 0.68 | 21.87 |
| 2 | *Agrocharis* *melanantha* Hochst. | 62 | 35 | 0.55 | 21.87 |
| 3 | *Agrostis* *gracilifolia* C. E. Hubb | 96 | 42 | 0.86 | 26.25 |
| 4 | *Agrostis* *quinqueseta* (Hochst. ex Steud.) Hochst. | 144 | 53 | 1.29 | 33.13 |
| 5 | *Alchemilla* *abyssinica* Fresen*.* | 577 | 94 | 5.19 | 58.75 |
| 6 | *Alchemilla* *ellenbecki* Engl*.* | 610 | 129 | 5.49 | 80.63 |
| 7 | *Alchemilla* *kiwuensis* Engl. | 144 | 58 | 1.29 | 36.25 |
| 8 | *Anchusa* *affinis* R.Br. ex DC. | 46 | 21 | 0.41 | 13.23 |
| 9 | *Andropogon* *lima* (Hack.) Stapf | 62 | 31 | 0.55 | 19.38 |
| 10 | *Andropogon* *amethystinus* Steud. | 61 | 25 | 0.54 | 15.63 |
| 11 | *Anthemis* *tigreensis* J. Gay ex A*.* Rich*.* | 128 | 50 | 0.15 | 31.25 |
| 12 | *Argyrolobium* *ramosissimum* Bak*.* | 71 | 35 | 0.63 | 44.38 |
| 13 | *Argyrolobium* *rupestre* (E. Mey.) Walp. | 33 | 18 | 0.29 | 11.25 |
| 14 | *Artemisia* *abyssinica* Sch. Bip. ex A*.* Rich. | 23 | 12 | 0.20 | 7.50 |
| 15 | *Bartsia* *longiflora* Hochst.ex Benth. | 5 | 3 | 0.04 | 1.88 |
| 16 | *Carduus* *schimperi* Sch. Bip. ex A. Rich. | 10 | 5 | 0.09 | 3.13 |
| 17 | *Carex* *conferta* Hochst*.* ex A. Rich | 5 | 2 | 0.04 | 1.25 |
| 18 | *Carex* *monostachya* A. Rich*.* | 29 | 10 | 0.26 | 6.25 |
| 19 | *Cineraria* *abyssinica* Sch. Bip. ex A. Rich. | 98 | 33 | 0.88 | 20.63 |
| 20 | *Conyza* *pyrrhopappa* Sch. Bip. ex A. Rich. | 21 | 11 | 0.18 | 6.88 |
| 21 | *Conyza* *stricta* Willd | 17 | 7 | 0.15 | 4.38 |
| 22 | *Crassula* *alba* Forssk. | 13 | 7 | 0.11 | 4.38 |
| 23 | *Cynoglosum* *amplifolium* Hochst. ex A. DC. | 41 | 23 | 0.36 | 14.38 |
| 24 | *Cynoglosum* *coeruleum* Hochst. ex A. DC. | 46 | 19 | 0.41 | 11.88 |
| 25 | *Cyperus* *elegantulus* Steud. | 172 | 46 | 1.54 | 28.75 |
| 26 | *Cyperus* *rigidifolius* Steud. | 146 | 42 | 1.31 | 26.25 |
| 27 | *Dicrocephala* *chrysanthemifolia* DC. | 11 | 5 | 0.09 | 3.13 |
| 28 | *Dipsacus pinnatifidus* Steud. ex A. Rich. | 24 | 9 | 0.21 | 5.63 |
| 29 | *Epilobium stereophyllum* Fresen. | 14 | 8 | 0.12 | 5.00 |
| 30 | *Erica arborea* L. | 20 | 11 | 0.18 | 6.88 |
| 31 | *Erica tenuipilosa* (Engl. ex Alm & Fries) Cheek | 2 | 2 | 0.01 | 1.25 |
| 32 | *Erigeron alpinus* L. | 10 | 6 | 0.09 | 3.75 |
| 33 | *Euryops pinifolius* A. Rich. | 702 | 104 | 6.32 | 65.00 |
| 34 | *Festuca abyssinica,* Hochst. ex A. Rich. | 46 | 22 | 0.41 | 13.75 |
| 35 | *Festuca macrophylla* Hochst. ex A. Rich. | 357 | 110 | 3.21 | 68.75 |
| 36 | *Festuca richardii* E.B.Alexeev | 1 | 1 | 0.01 | 0.63 |
| 37 | *Galium simense* Fresen. | 12 | 6 | 0.10 | 3.75 |
| 38 | *Hebenstretia angolensis* Rolfe. | 9 | 5 | 0.08 | 3.13 |
| 39 | *Hedbergia abyssinica* (Hochst. ex Benth. | 2 | 2 | 0.01 | 1.25 |
| 40 | *Helichrysum formosissimum* Sch. Bip. ex A. Rich. | 9 | 5 | 0.08 | 3.13 |
| 41 | *Helichrysum stenopterum* DC. | 18 | 9 | 0.16 | 5.63 |
| 42 | *Helichrysum forsskahlii* (J.F. Gmel.) Hilliard& Burtt | 17 | 8 | 0.15 | 5.00 |
| 43 | *Helichrysum splendidum* (Thumb.) Less | 1142 | 147 | 10.28 | 91.88 |
| 44 | *Helictotrichon elongatum* (Hochst. ex. A. Rich.) C. E. Hubb. | 11 | 6 | 0.09 | 3.75 |
| 45 | *Hesperantha petitiana* (A. Rich.) Baker | 10 | 6 | 0.09 | 3.75 |
| 46 | *Hypericum revolutum* Vahl | 42 | 20 | 0.37 | 12.50 |
| 47 | *Isolepis costata* A. Rich. | 12 | 8 | 0.10 | 5.00 |
| 48 | *kalanchoe petitiana* A. Rich. | 18 | 10 | 0.16 | 6.25 |
| 49 | *Kniphofia foliosa* Hochst. | 1 | 1 | 0.01 | 0.63 |
| 50 | *Lobelia rhynchopetalum* Hemsl. | 1 | 1 | 0.01 | 0.63 |
| 51 | *Luzula abyssinica* Parl. | 3 | 3 | 0.02 | 1.88 |
| 52 | *Nepeta azurea* R.Br. ex Benth. | 2 | 2 | 0.01 | 1.25 |
| 53 | *Pimpinella oreophila* Hook. | 3 | 2 | 0.02 | 1.25 |
| 54 | *Plectocephalus varians* (A.Rich.) C. Jeffrey ex. Cufod. | 3 | 2 | 0.02 | 1.25 |
| 55 | *Ranunculus multifidus* Forssk. | 6 | 2 | 0.05 | 1.25 |
| 56 | *Rhabdotosperma scrophularifolia* (Hochst. ex A. Rich.) Hartle | 4 | 3 | 0.03 | 1.88 |
| 57 | *Rubus volkensii* Engl. | 6 | 5 | 0.05 | 3.13 |
| 58 | *Rumex abyssinicus* Jacq. | 3 | 3 | 0.02 | 1.88 |
| 59 | *Rumex nepalensis* Spreng. | 2 | 2 | 0.01 | 1.25 |
| 60 | *Rytidosperma subulata* (A. Rich.) Cope | 3 | 2 | 0.02 | 1.25 |
| 61 | *Salvia merjamie* Forssk. | 5 | 4 | 0.04 | 2.50 |
| 62 | *Satureja pseudosimensis* Brenan | 3 | 2 | 0.02 | 1.25 |
| 63 | *Scabiosa columbaria* L. | 3 | 2 | 0.02 | 1.25 |
| 64 | *Senecio ragazi* Chiov. | 3 | 2 | 0.02 | 1.25 |
| 65 | *Senecio schulzii* Hochst. ex. A. Rich. | 3 | 2 | 0.02 | 1.25 |
| 66 | *Senecio steudelii* Sch. Bip. ex A. Rich. | 5 | 3 | 0.04 | 1.88 |
| 67 | *Senecio subsessilis* Oliv. & Hiern | 2 | 2 | 0.01 | 1.25 |
| 68 | *Swertia kilimandscharica* Engl. | 8 | 8 | 0.07 | 5.00 |
| 69 | *Thymus* *schimperi* Ronniger | 1466 | 160 | 13.20 | 100 |
| 70 | *Trifolium polystachyum* Fresen. | 847 | 142 | 7.63 | 88.75 |
| 71 | *Trifolium usambarense* Taub. | 753 | 144 | 6.78 | 90.00 |
| 72 | *Urtica simensis* Steudel | 336 | 79 | 3.02 | 49.38 |
| 73 | *Verbascum sinaiticum* Benth. | 10 | 4 | 0.09 | 2.50 |
| 74 | *Veronica glandulosa* Hochst .ex Benth. | 8 | 4 | 0.07 | 2.50 |
